# Trends in Uveal Melanoma Presentation and Survival During Five Decades: A Nationwide Survey of 3898 Swedish Patients

**DOI:** 10.3389/fmed.2022.926034

**Published:** 2022-06-02

**Authors:** Viktor Gill, Christina Herrspiegel, Shiva Sabazade, Maria Fili, Louise Bergman, Bertil Damato, Stefan Seregard, Gustav Stålhammar

**Affiliations:** ^1^Department of Pathology, Västmanland Hospital Västerås, Västerås, Sweden; ^2^Division of Eye and Vision, Department of Clinical Neuroscience, Karolinska Institutet, Stockholm, Sweden; ^3^St. Erik Eye Hospital, Stockholm, Sweden; ^4^Ocular Oncology Service, Moorfields Eye Hospital, London, United Kingdom; ^5^Nuffield Laboratory of Ophthalmology, Nuffield Department of Clinical Neurosciences, University of Oxford, Oxford, United Kingdom

**Keywords:** uveal melanoma, survival, treatment, time-trend, ophthalmology (MeSH), cancer, melanoma, Sweden

## Abstract

**Background:**

In contrast to most other cancers, uveal melanoma (UM) is characterized by an absence of major improvements in patient survival during the last several decades. In this study, we examine changes in incidence rates, patient age and tumor size at diagnosis, treatment practices and survival for patients diagnosed in Sweden during the period 1960–2010.

**Methods:**

All patients diagnosed with posterior UM between January 1st, 1960, and December 31st, 2009, in Sweden, were included (*n* = 3898). Trends in incidence, primary treatment modality, patient age and tumor size were analyzed. Disease-specific survival was plotted in Kaplan–Meier curves and the cumulative incidence of UM-related mortality was evaluated in competing risk analysis.

**Results:**

Crude (6.5–11.6 cases/million/year) and age-standardized incidence rates (5.6–9.6 cases/million/year) varied between individual years during the study period, but both had a stable linear trend overall (*p* ≥ 0.12). Gradually, plaque brachytherapy with ruthenium-106 replaced enucleation as the most common primary treatment. The mean patient age at diagnosis increased from 59.8 years in 1960 to 66.0 in 2009. Conversely, the mean tumor size became gradually smaller during the period. In linear regression, the basal diameter and tumor apical thickness decreased with a slope coefficient of −0.03 mm (*p* = 0.012) and −0.05 mm (*p* = 1.2 × 10^–5^) per year after 1960, respectively. Patients diagnosed after 1990 had significantly better disease-specific survival than patients diagnosed before 1990 (*p* = 2.0 × 10^–17^). Similarly, the cumulative incidence of UM-related mortality was highest for patients diagnosed 1960–1969 and 1970–1979, with slightly lower incidences for patients diagnosed 1980–1989 and even lower for those diagnosed after 1990 (*p* = 7.1 × 10^–13^). The incidence of mortality from other causes than UM did not differ between periods (*p* = 0.16).

**Conclusion:**

In the period from 1960–2010, crude and age-standardized incidence rates of UM have remained stable in Sweden. Several other aspects have changed: Plaque brachytherapy with ruthenium-106 has replaced enucleation as the most common primary treatment modality; patients have become older and their tumors smaller at the time of diagnosis; and their survival has improved. This might indicate a beneficial survival effect of earlier diagnosis and treatment, but the potential influence from lead-time bias should be taken into consideration.

## Introduction

Within 10 years from diagnosis, 28–40% of patients diagnosed with uveal melanoma (UM) succumb to metastatic disease, and the relative survival has been estimated to be 66% (1–3). Although very few patients have detectable metastases at presentation, and virtually all undergo primary tumor treatment, it remains debated if primary tumor treatment has any effect on patient survival (4, 5). Calculations based on observed tumor doubling times indicate that systemic micrometastases could be present well before the primary tumor is detected (6–8). However, a recent meta-analysis of patients who underwent primary tumor treatment more than 5 years after diagnosis showed that 80–90% developed metastases, compared to the 35–50% of patients who received primary treatment, which suggests that there might be a beneficial therapeutic effect on survival after all (9).

Similarly, no effective treatment has been available once radiologically detectable metastases have developed (10, 11). Previous therapeutic regimes for metastatic disease include partial hepatectomy, conventional chemotherapy, chemoimmunotherapy, hepatic intra-arterial chemotherapy and transarterial chemoembolization. Recently, encouraging results were presented when the bispecific fusion protein tebentafusp was shown to prolong median overall survival from 16 to 22 months in a group of previously untreated HLA- A*02:01–positive patients with metastatic UM (12). This drug has now been approved by the U.S. Food and Drug Administration (FDA).

Several studies have reported no significant improvement in relative or disease-specific survival rates over the last several decades (13–17). However, at least two exceptions exist: a Danish study including patients diagnosed between 1943 and 2021, which showed improving relative survival rates and decreasing primary tumor size at diagnosis over time (18); and a study from St. Erik Eye Hospital, which indicated that patients diagnosed between 1990 and 1998 had better relative survival than patients diagnosed between 1960 and 1969 (19).

In this national retrospective cohort, we aimed to extend previous studies and determine the incidence and competing-risk survival with data from two additional decades. We also investigated patient age and tumor size at diagnosis as well as ocular treatment type.

## Materials and Methods

This study was approved by the Swedish Ethical Review Authority (reference 2020-02835) and adhered to the tenets of the Declaration of Helsinki. All patients residing in Sweden who were diagnosed with posterior UM (i.e., originating in the choroid or the ciliary body) between January 1st, 1960, and December 31st, 2009, were included (*n* = 3898). The data for the years 1960–1979 was based on a national survey that included reports from the Swedish Cancer Registry and hospital files. For the years 1980–2009, the data was collected from the digitalized treatment registry at St. Erik Eye Hospital according to a previously described methodology (20–22). Since 1960, all Swedish UM patients have been diagnosed at St. Erik Eye Hospital and its predecessor Karolinska University Hospital, with very few exceptions. In cases with very large tumors that have been immediately enucleated in the home clinic, the eyes have been sent for histological examination at the St. Erik Ophthalmic Pathology Laboratory. Parts of this data has been published previously and it has been estimated that the survey captured more than 95% of the UM cases in the country (19, 23). Data from the Cause of Death Registry was automatically fed to the treatment registry to achieve a similar degree of capture as the survey. Thus, all data for this study had been made available previously and no additional collection of patient names, personal identification numbers, diagnoses, treatments, photographs, contact information or any other data that could be traced back to any individual was required. The Cause of Death Registry reported the underlying cause of death, according to the International Classification of Diseases (ICD) code in use at the time of the death. The method of establishing the cause of death was coded as autopsy, clinical examination or forensic investigation. The Cause of Death Registry had been cross-referenced with data from patient medical records so that misclassifications of death from UM as death from cutaneous melanoma were corrected. Data on the number of metastases and affected organs is not available. Similarly, we have no access to data on metastatic treatment for the included patients. UM diagnoses were primarily established upon histological examination of enucleated eyes throughout the study period. For the smaller proportions of patients that underwent eye-sparing plaque brachytherapy, the diagnosis was based on clinical examination with a slit-lamp biomicroscope aided by serial fundus photography, ultrasonography, fluorescein angiography and optical coherence tomography (OCT) as needed and after each respective technique has become available. Tumor dimensions (largest basal diameter and apical thickness) were measured in freshly enucleated specimens before paraffin embedding. In case of eye-preserving treatment, tumor diameters were estimated upon slit-lamp biomicroscopy and with fundus photographs, aided by reference points such as the optic disc. The introduction of plaque brachytherapy in 1979 largely coincided with the introduction of A and B-scan ultrasonography for measurements of tumor thickness.

### Statistical Analyses

*P*-values below 0.05 were considered statistically significant, all *p*-values being two-sided. Files from Statistics Sweden including records from the Swedish population censuses of 1960 through 1998 were used for calculation of incidence rates (24). Before 1970, the statistics were incomplete regarding the age distribution, preventing analysis for the period 1960–1969. Age-standardization of incidence numbers over the study period was performed by a direct method, with the Swedish population during the period 1970–1974 taken as a standard, according to the stratum weights (Table 1). The relative change in incidence over the period was calculated by linear regression after logarithmic transformation of incidence data. The cumulative incidence of UM-related mortality was plotted in cumulative incidence function estimates from competing risks data with the cmprsk package for R (SurvComp, RRID:SCR_003054), and the equality of survival distributions was tested with Gray’s test for equality (25). Disease-specific survival was plotted in Kaplan–Meier curves and the Wilcoxon (Gehan) test applied. All statistical analyses except competing risk analyses were performed using IBM SPSS statistics version 27 (Armonk, NY, United States, RRID:SCR_016479).

**TABLE 1:** Stratum weights of the Swedish standard population 1970–1974.

Age group (years)	Stratum weight
0–24	0.35
25–34	0.15
35–44	0.11
45–54	0.13
55–64	0.12
65–74	0.09
75–84	0.05
> 85	0.01

## Results

### Descriptive Statistics

A total of 3,898 patients had been diagnosed with UM between January 1st, 1960, and December 31st, 2009. Their tumors had a mean thickness and diameter of 6.1 and 11.4 mm, respectively. In terms of primary treatment, 904 patients (23%) had been treated with ruthenium-106 plaque brachytherapy, 139 (4%) with iodine-125 brachytherapy and 2,846 (73%) by enucleation. Of the 3898 included patients, 2,511 had died by the time of data collection. Median follow-up for the 1387 survivors was 12.6 years (interquartile range 11.1, Table 2).

**TABLE 2:** Demographics and clinical features of study patients and tumors.

*n*	3898
Mean age at diagnosis, years (SD)	62.6 (13.6)
Mean tumor thickness, mm (SD)	6.1 (3.1)
Mean tumor diameter, mm (SD)	11.4 (4.1)
Primary treatment, *n* (%)	
^106^Ru brachytherapy	904 (23)
^125^I brachytherapy	139 (4)
Enucleation	2846 (73)
Diagnosis period, *n* (%)	
1960–1969	807 (21)
1970–1979	755 (19)
1980–1989	753 (19)
1990–1999	811 (21)
2000–2009	772 (20)
Follow-up years, median*^a^* (IQR)	12.6 (11.1)

*SD, Standard deviation. ^106^Ru, Ruthenium-106. ^125^I, Iodine-125. IQR, Interquartile range. ^a^For survivors.*

### Changes in Treatment Over Time

**FIGURE 1:**
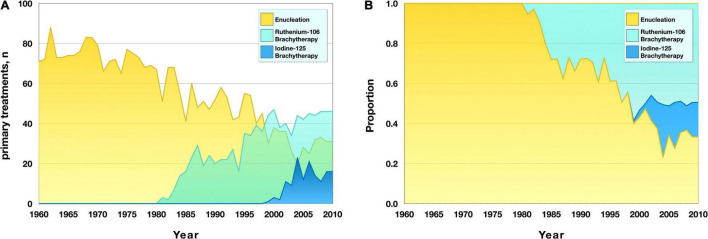
Primary treatment modality for uveal melanoma in the period 1960–2010. **(A)** Number of treatments with the respective modality. Plaque brachytherapy with ruthenium-106 was introduced in 1979 and plaque brachytherapy with iodine-125 was introduced in 1999. **(B)** Proportion of all tumors treated with the respective modality.

Before 1979, enucleation was the only primary treatment in use. In late 1979, the first patient was treated with ruthenium-106 plaque brachytherapy. Gradually, the latter has become the mainstay treatment. Since 1999, plaque brachytherapy with ruthenium-106 has been the most common primary treatment for UM. This year also marked the introduction of iodine-125 plaque brachytherapy, which is reserved for tumors with an apex height of more than 6 or 7 mm ever since (22). In 2009, primary treatment modalities in reducing order of frequency were (1) ruthenium-106 brachytherapy, (2) enucleation, and (3) iodine-125 brachytherapy (Figure 1).

### Changes in Patient Age and Tumor Size at Diagnosis

**FIGURE 2:**
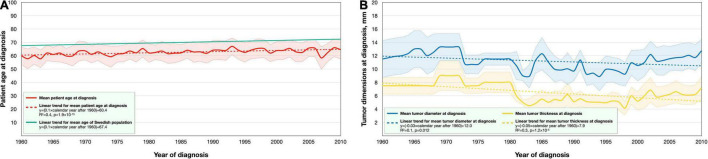
Mean patient age and tumor dimensions at diagnosis in the period 1960–2010. **(A)** Uveal melanoma patients were gradually older at diagnosis during the period (red). The linear trend for their mean age (dashed red) increased with 0.1 years per calendar year, which was similar to the linear trend for the mean age of the Swedish population (green). **(B)** Reversely, the mean largest basal tumor diameter (blue) and mean tumor apical thickness (yellow) were gradually smaller at diagnosis, with linear trends (dashed) indicating a decline of 0.03 and 0.05 mm per calendar year, respectively.

Patient age at diagnosis gradually increased throughout the study period, from a mean of 59.8 years (SD 13.1) in 1960 to a mean of 66.0 years (SD 13.5) in 2009. In linear regression, patients’ age at diagnosis increased with a slope coefficient of 0.1 per year after 1960. The patients’ age at diagnosis was fitted to a linear function (*R*^2^ = 0.4, *p* = 1.9 × 10^–15^), where *x* was the consecutive year after 1960: number of specimens = (0.09*x*) + 60.4. The slope coefficient was similar to the increase of the Swedish population = (0.1*x*) + 67.4 (Figure 2A).

Conversely, the largest basal tumor diameter and apical thickness gradually diminished during the period. In linear regression, the basal diameter decreased with a slope coefficient of −0.03 mm per year after 1960 (*R*^2^ = 0.1, *p* = 0.012). Tumor thickness decreased with a slope coefficient of −0.05 mm per year (*R*^2^ = 0.3, *p* = 1.2 × 10^–5^, Figure 2B).

### Crude and Age-Standardized Incidence Rates

**FIGURE 3:**
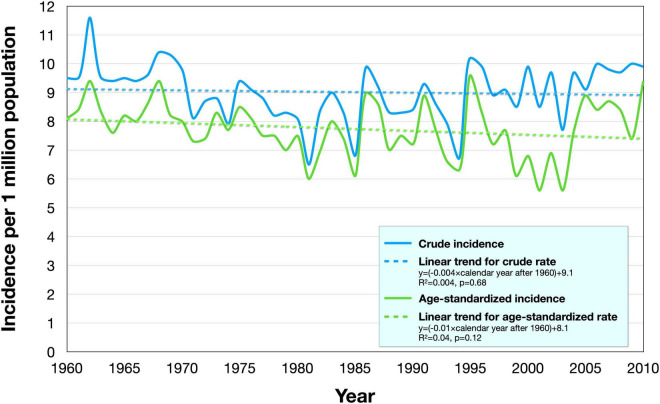
Incidence rates in the period 1960–2010. The crude (blue) and age-standardized (green) incidence rates varied between 6.5 to 11.6 cases/million/year, and 5.6–9.6 cases/million/year, respectively. In linear regression (dashed), neither crude nor age-standardized incidence rates changed significantly over time.

Between 1960 and 2010, the crude incidence of UM in the Swedish population was 6.5–11.6 cases/million/year, and the age-standardized incidence rate was 5.6–9.6 cases/million/year (Table 3). In linear regression, neither crude nor age-standardized incidence rates changed significantly over time (*p* = 0.68 and *p* = 0.12, respectively, Figure 3).

**TABLE 3:** Crude and age-standardized incidence rates of uveal melanoma (UM) in the period 1960–2010.

Year	Swedish population, n	Mean age of uveal melanoma patients at diagnosis	Mean age in Swedish population	Number of new UM cases	Crude incidence rate/million/year	Age-standardized incidence rater/million/year
1960	7497967	60	66	71	9.5	8.1
1961	7556293	58	66	72	9.5	8.4
1962	7614619	60	66	88	11.6	9.4
1963	7672945	58	66	73	9.5	8.3
1964	7731271	62	66	73	9.4	7.6
1965	7789597	61	66	74	9.5	8.2
1966	7847923	62	66	74	9.4	8.0
1967	7906249	60	66	76	9.6	8.6
1968	7964575	60	66	83	10.4	9.4
1969	8022901	64	66	83	10.3	8.2
1970	8081229	64	70	79	9.8	8.0
1971	8106671	61	71	66	8.1	7.3
1972	8132113	63	71	71	8.7	7.4
1973	8157555	61	71	72	8.8	8.3
1974	8182997	59	71	65	7.9	7.7
1975	8208442	61	72	77	9.4	8.5
1976	8230341	62	72	75	9.1	8.1
1977	8252240	64	72	73	8.8	7.5
1978	8274139	62	73	68	8.2	7.5
1979	8296038	65	73	69	8.3	7.0
1980	8317937	62	69	67	8.1	7.5
1981	8323033	63	69	54	6.5	6.0
1982	8327484	64	69	70	8.4	7.0
1983	8330573	62	69	75	9.0	8.0
1984	8342621	62	69	69	8.3	7.4
1985	8358139	62	69	57	6.8	6.1
1986	8381515	64	69	83	9.9	9.0
1987	8414083	63	69	77	9.2	8.6
1988	8458888	63	69	70	8.3	7.0
1989	8527036	62	69	71	8.3	7.5
1990	8590630	64	71	72	8.4	7.2
1991	8644119	64	72	80	9.3	8.9
1992	8692013	64	72	75	8.6	7.7
1993	8745109	67	72	69	7.9	6.6
1994	8816381	64	72	59	6.7	6.3
1995	8837496	63	72	90	10.2	9.6
1996	8844499	64	72	88	9.9	8.3
1997	8847625	66	72	79	8.9	7.2
1998	8854322	65	72	81	9.1	7.7
1999	8861426	62	72	75	8.5	6.1
2000	8882792	64	71	88	9.9	6.8
2001	8909128	64	71	76	8.5	5.6
2002	8940788	66	71	87	9.7	6.9
2003	8975670	63	71	69	7.7	5.6
2004	9011392	63	71	87	9.7	7.7
2005	9047752	66	71	82	9.1	8.9
2006	9113257	66	71	91	10.0	8.4
2007	9182927	58	71	90	9.8	8.7
2008	9256347	62	71	90	9.7	8.4
2009	9340682	66	71	93	10.0	7.4
2010	9415570	65	72	93	9.9	9.4

### Survival

**FIGURE 4:**
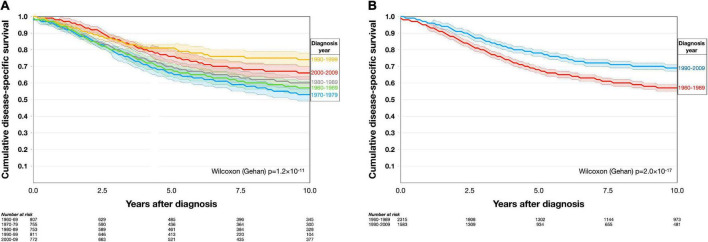
Kaplan–Meier disease-specific survival in the period 1960–2010. **(A)** Patients diagnosed in different decennia had dissimilar disease-specific survival. **(B)** Patients diagnosed after 1990 had significantly better disease-specific survival than patients diagnosed before 1990. Colored areas represent 95% confidence intervals.

With Kaplan–Meier analysis, patient survival changed over time between 1960 and 2010 [Wilcoxon (Gehan) *p* = 1.2 × 10^–11^, Figure 4A]. Patients diagnosed after 1990 had significantly better disease-specific survival than patients diagnosed before 1990 (*p* = 2.0 × 10^–17^, Figure 4B).

**FIGURE 5:**
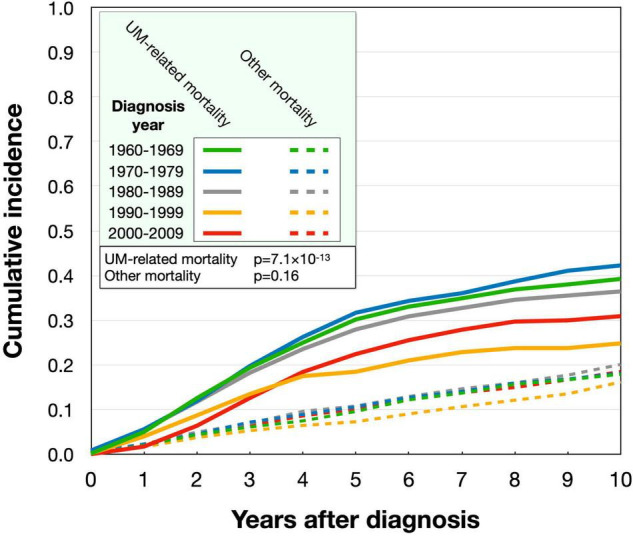
Cumulative incidence of uveal melanoma-related mortality and mortality from other causes in the period 1960–2010. The uveal melanoma-related mortality was highest for patients diagnosed 1960–1969 and 1970–1979, with slightly lower incidences 1980–1989 and even lower after 1990. The incidence of mortality from other causes than uveal melanoma (dashed) did not differ between the periods.

Similarly, the cumulative incidence of UM-related mortality was highest in patients diagnosed between 1960 and 1979, with a slightly lower mortality in patients diagnosed between 1980 and 1989 and even lower mortality in those diagnosed after 1990 (Gray’s test for equality *p* = 7.1 × 10^–13^). The mortality from other causes than UM did not change between the periods (*p* = 0.16, Figure 5).

## Discussion

In this study, we found that the age of UM patients at diagnosis increased in the period from 1960–2010, in line with the yearly increase in the mean age of the Swedish population. Conversely, tumor size decreased slightly during this period, whereas crude and age-standardized incidence rates remained stable. Ruthenium-106 plaque brachytherapy replaced enucleation as the most common primary treatment modality. Most importantly, Kaplan–Meier disease specific survival improved, as did the cumulative incidence of UM-related mortality.

The improvement in survival could be related to the decreasing tumor size (26–28). In contrast to cutaneous melanoma, UM is characterized by a low response rate to checkpoint inhibitors and absence of druggable mutations in receptor tyrosine kinases, and recent breakthroughs in the treatment of metastatic UM has not been introduced in our clinical routine at St. Erik Eye Hospital and is not reflected in our analyzed data (10, 12, 29–31). Possible reasons for the decreasing tumor size at diagnosis include the introduction of widespread diabetic retinopathy screening and increasing frequency of cataract surgery. Diagnosis and treatment of smaller tumors may have introduced bias by increasing misdiagnosis of choroidal nevi as small melanomas (false positives); however, the incidence of UM diagnosis did not increase as would have occurred if the number of false positives had increased. We therefore interpret these results as an indication that earlier treatment of UM leads to improved patient survival. To some extent, the results may be due to lead time bias, that is, time is added to a patient’s survival because of earlier diagnosis rather than postponed time of death (32). Our interpretation may also be considered controversial as the impact of primary tumor treatment on patient prognosis is debated (4, 33). The recent observation of a 80–90% metastatic rate among patients with untreated primary tumors indicates that a beneficial effect might exist after all (9). This may be further corroborated by the observation that there are no survival differences between melanomas originating in the choroid or the iris when adjusting for tumor size, although additional studies are needed to verify this observation (34). The reason for the relatively good prognosis in iris melanoma is likely that they are typically diagnosed at a small size. All small tumors may not necessarily grow large and acquire monosomy 3, *BAP1* mutations, vasculogenic mimicry or other high-risk features even if left untreated (4). Some, but not all, tumors seem to have these features from the outset, and there is marked variability in their growth rate and considerable intratumor heterogeneity of various risk factors (8, 35–39). Nonetheless, the likelihood of high-risk features increases with increasing tumor size (8, 40–42). Consequently, if tumors are smaller at treatment, a greater proportion of tumors that otherwise would have developed high-risk features at a later stage would be included, thus reducing metastatic rates overall (43).

A general assumption is that the progression from malignant transformation of a melanocytic nevus in the choroid to growth of systemic UM macrometastases is very slow, and that older patients have more long-standing, and often larger, tumors with increased risk for metastasis (8, 44, 45). In a publication by Damato et al. from 2014, younger patients had smaller tumors, lower TNM stage, lower frequency of ciliary body involvement and monosomy 3 (43). Our inverse relationship between gradually older patients and gradually smaller tumors is therefore not intuitive, but replicates what has been observed previously in Denmark (18). We would encourage further similar examinations in other cohorts over long periods of time to shed further light on this phenomenon.

In two previous publications from our institution at St. Erik eye Hospital, the incidence of UM has declined significantly for men but not women between 1960 and 1968, with the relative survival rates for both sexes improving significantly (19, 23). There was a slight shift in the curves with a tendency for increasing incidence, tumor size and mortality in the last decade, but we are content that the overall trend remains intact and that patients diagnosed in the last decade still had lower incidence of UM-related mortality than patients diagnosed during any period prior to 1990.

Limitations to this study include the retrospective analysis with limited control over confounding factors. The many factors that influence survival, which include *BAP1* mutations, gene expression class, ciliary body involvement and presence of vasculogenic mimicry, were not accounted for and neither do we know how these factors were distributed in the different time periods analyzed. Secondly, estimations of disease-specific survival and the incidence of UM-related mortality rely on correct diagnosis of the cause of death, and surveys and treatment registries are not always accurate in this regard. The number of misclassifications should be reduced by cross-reference between the treatment registry and medical journals, but we cannot exclude that some UM-related deaths were coded as death from other causes and vice versa. Thirdly, we had no access to data on metastatic profiles or treatments, which would have provided important clues to changes in patient survival. Lastly, the Kaplan–Meier method may be influenced by competing risks (i.e., death from other causes than UM). The cumulative incidence of UM-related mortality in competing risk analysis is not subject to such bias.

In conclusion, this study provides further evidence that earlier ocular treatment of UM prevents metastatic death in some patients. We encourage further research to verify and clarify the observation of an inverse relationship between patient age and tumor size and the influence of lead time bias on the improved survival rates.

## Resource Identification Initiative

The cumulative incidence of UM-related mortality was plotted in cumulative incidence function estimates from competing risks data with the cmprsk package for R (SurvComp, RRID:SCR_003054). All other statistical analyses were performed using IBM SPSS statistics version 27 (Armonk, NY, United States, RRID:SCR_016479).

## Data Availability Statement

The data analyzed in this study is subject to the following licenses/restrictions: Anonymization is required. No patient names, personal identifiers, addresses, other contact details, photographs, or dates of diagnosis, treatment, follow-up or death may be shared. Requests to access these datasets should be directed to GS, Gustav.Stalhammar@ki.se.

## Ethics Statement

The studies involving human participants were reviewed and approved by the Swedish Ethical Review Authority (reference 2020-02835). Written informed consent for participation was not required for this study in accordance with the national legislation and the institutional requirements.

## Author Contributions

VG, CH, and ShS: writing – original draft. MF and LB: investigation and writing – review and editing. BD: writing – review and editing. StS: methodology and writing – review and editing. GS: conceptualization, formal analysis, resources, visualization, supervision, project administration, and funding acquisition. All authors contributed to the article and approved the submitted version.

## Conflict of Interest

The authors declare that the research was conducted in the absence of any commercial or financial relationships that could be construed as a potential conflict of interest.

## Publisher’s Note

All claims expressed in this article are solely those of the authors and do not necessarily represent those of their affiliated organizations, or those of the publisher, the editors and the reviewers. Any product that may be evaluated in this article, or claim that may be made by its manufacturer, is not guaranteed or endorsed by the publisher.
